# Time Course Study of Blood Pressure in Term and Preterm Infants Immediately after Birth

**DOI:** 10.1371/journal.pone.0114504

**Published:** 2014-12-16

**Authors:** Gerhard Pichler, Po-Yin Cheung, Corinna Binder, Megan O’Reilly, Bernhard Schwaberger, Khalid Aziz, Berndt Urlesberger, Georg M. Schmölzer

**Affiliations:** 1 Centre for the Studies of Asphyxia and Resuscitation, Neonatal Research Unit, Royal Alexandra Hospital, Edmonton, Canada; 2 Department of Pediatrics, University of Alberta, Edmonton, Canada; 3 Division of Neonatology, Department of Pediatrics, Medical University, Graz, Austria; National Taiwan University Hospital, Taiwan

## Abstract

**Objective:**

To describe temporal changes in systolic, diastolic, and mean blood pressure (SBP, DBP, and MBP, respectively) in term and preterm infants immediately after birth.

**Methods:**

Prospective observational two-center study. In term infants SBP, DBP, and MBP were assessed non-invasively every minute for the first 15 minutes, and in preterm infants every minute for the first 15 minutes, as well as at 20, 25, 30, 45, and 60 minutes after birth. Regression analyses were performed by gender and respiratory support in all neonates; and by mode of delivery, cord clamping time, and development of ultrasound-detected brain injury in preterm neonates.

**Results:**

Term infants (n = 54) had a mean (SD) birth weight of 3298 (442) g and gestational age of 38 (1) weeks, and preterm infants (n = 94) weighed 1340 (672) g and were 30 (3) weeks gestation. Term infants’ SBP, DBP and MBP within the first 15 minutes after birth were independent of gender or respiratory support. Linear mixed regression analysis showed that preterm infants, who were female, born vaginally, had delayed cord clamping and did not require positive pressure ventilation nor develop periventricular injury or ventriculomegaly, had significantly higher SBP, DBP, and MBP at some measurement points within the first hour after birth.

**Conclusions:**

We present novel reference ranges of BP immediately after birth in a cohort of term and preterm neonates. They may aid in optimization of cardiovascular support during early transition at all gestations.

## Introduction

Throughout fetal life pulmonary vascular resistance remains high and the majority of right ventricular output bypasses the lungs through the ductus arteriosus into the systemic circulation [1]–[3]. Following the elimination of the low-resistance placenta and the reduction of pulmonary vascular resistance with the first breaths after birth, there is a transition from fetal to neonatal circulation and major hemodynamic changes occur. Pulmonary vascular resistance drops, systemic vascular resistance (SVR) increases, systemic blood flow is directed through the lungs, and within minutes to hours ductal shunt reverses, with complete physiologic closure of the ductus arteriosus within 48–72 hours after birth in healthy, term infants [1]–[5]. Blood pressure (BP) is a composite of cardiac function and peripheral circulation as determined by cardiac output (CO) and SVR, respectively. BP is usually monitored continuously in critically ill neonates using an indwelling arterial catheter [6] However, invasive BP monitoring is not available immediately after birth (or at all in healthy infants). Consequently, non-invasive BP monitoring is commonly used for cardiovascular assessment. There are no available reference values for BP in the first few minutes after birth.

This study aims to define reference ranges for systolic BP (SBP), diastolic BP (DBP) and mean BP (MBP) in the first hour after birth in preterm infants and in the first 15 minutes in term infants and to formulate percentile charts for use after delivery. We further examine differences in the temporal patterns of BP with respect to gender, mode of delivery, delayed cord clamping (DCC), gestational age, level of respiratory support, and development of severe cerebral ultrasound abnormalities.

## Methods

This two-center prospective observational study was carried out at the Royal Alexandra Hospital, Edmonton, Canada and the Department of Pediatrics, Medical University Graz, Austria, both tertiary perinatal centers with approximately 6000 and 3000 births per year and admitting approximately 350 and 100 infants with a birth weight of <1500 g to the neonatal nursery annually, respectively. The Royal Alexandra Hospital Research Committee and Health Ethics Research Board, University of Alberta and the Health Ethics Research Board, Medical University Graz approved. Written parental consent was obtained in all neonates: in Edmonton before or after birth (deferred consent) in Graz only before birth, if possible 24 hours before birth.” Term infants born after elective cesarean section (in Graz only) and preterm infants (from both centers) born either vaginally or by caesarean section were included in Graz and in Edmonton between February 2013 and August 2013. Only neonates without history of blood loss or of cardiovascular support within the first hour after birth were included. The same protocol in both institutions was used. The research team, who was not involved in direct clinical care, attended all included deliveries. All preterm infants were stabilized for the first hour in the delivery room before admission to the Neonatal Intensive Care Unit.

### Monitoring systems and data collection

IntelliVue MP50 monitors (Philips Healthcare, Philips Electronics Ltd., Markham, ON, Canada) were used to continuously measure heart rate, oxygen saturation, and blood pressure (BP). BP was measured using a non-invasive BP cuff of appropriate size (#1, #2 or #3) on the left upper arm. Cuff size diameter was chosen according to the circumference of the infants left arm. The left upper arm was chosen to avoid interference with the pulse oximetery measurements at the infant’s right hand or wrist. In term infants BP measurements were started immediately after birth and performed at each minute until the infants were 15 minutes of age and brought to the father or mother for kangaroo-care. In preterm infants BP measurements were performed at each minute for the first 15 minutes, then at 20, 25, 30, 45, and 60 minutes after birth. In the Edmonton site, DCC was routinely performed for 60 seconds after delivery unless the obstetric team noted contraindications (e.g. antepartum hemorrhage, fetal/neonatal bradycardia or apnea) and clamped earlier. All parameters were stored continuously in a multichannel system “alpha-trace digital MM” (B.E.S.T. Medical Systems, Vienna, Austria) for subsequent analysis.

### Statistical analysis

Demographics of study infants were recorded. Centiles (10^th^, 25^th^, 50^th^, 75^th^, and 90^th^) for SBP, DBP and MBP were calculated by using the LMS-method described by Cole and Green and were fitted by using LMSchartmaker Version 2.54, a program to calculate age-related reference percentiles using the LMS method (Pan H, Cole TJ, http://www.healthforallchildren.co.uk/; 2011, Institute of Child Health, London, England). Subgroup analyses for term infants (gender, mode of delivery) and preterm infants were performed according to gender, gestational age (23^+0^–27^+6^ vs. 28^+0^–31^+6^ vs. 32^+0^–36^+6^), mode of delivery, DCC for 60 seconds versus earlier cord clamping <60 seconds (ECC), level of respiratory support applied with T-piece mask (continuous positive airway pressure (CPAP) vs. positive pressure ventilation (PPV)), cardiovascular support, and ultrasound detected preterm brain injury defined as intraventricular haemorrhage, parenchymal lesions and/or ventriculomegaly seen on cerebral ultrasound (performed on day 4±1, 8±1 and 14±1), which were defined prior the start of study. The data are presented as mean ±standard deviation (±SD) for normally distributed continuous variables and median (interquartile range, IQR) when the distribution was skewed. Data were compared using Student’s *t*-test and Mann-Whitney U test for parametric and nonparametric continuous variables, respectively, and χ^2^ for categorical variables. A linear mixed regression model with correction for gender, gestational age, mode of delivery, DCC or ECC, level of respiratory support (CPAP vs. PPV) was used for calculation of overall effects and differences between groups at each minute. p-values are 2-sided and p<0.05 was considered statistically significant. Subgroup analyses were considered in an explorative sense; therefore, no multiple testing corrections were performed. Statistical analyses were performed with Stata (Intercooled 10, Statacorp, Texas, USA).

## Results

**Figure 1:**
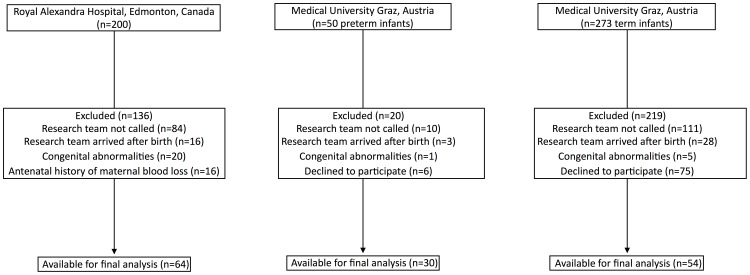
Flow diagram of eligible neonates.

Fifty-four term (Graz only) and 94 preterm infants (Edmonton n = 64, Graz n = 30,) were studied (Fig. 1) and their demographics are shown in Table 1. During the study period a total of 200 and 50 preterm infants were born in Edmonton and Graz, respectively. SBP, DBP, and MBP for term and preterm infants are presented in Tables 2–4.

**Table 1:** Clinical data of study infants.

	Term infants (n = 54)	Preterm infants (n = 94)	Preterm infants <28 weeks (n = 26)	Preterm infants 28^+0 −^31^+6^ weeks (n = 33)	Preterm infants >32 weeks (n = 35)
**Birth weight (g)^#^**	3230(3040–3464)	1400(830–1860)	760(650–830)	1280(960–1650)	1980(1670–2170)
**Gestational age (weeks)**	38±1	30±3	25±1	30±1	33±1
Male (n)*	27 (50%)	41 (44%)	11 (42%)	11 (33%)	14 (40%)
Any antenatal steroids (n)*	0 (0%)	69 (73%)	21 (80%)	30 (90%)	18 (51%)
Caesarean section*	54 (100%)	39 (72%)	20 (77%)	26 (78%)	24 (68%)
**Apgar score at 1 minute** ^#^	9 (9–9)	5 (2–7)	4 (1–5)	5 (4–7)	8 (5–8)
**Apgar score at 5 minutes** ^#^	10 (10–10)	7 (6–9)	6 (5–7)	7 (6–8)	9 (7–9)
**Apgar score at 10 minutes** ^#^	10 (10–10)	8 (7–9)	8 (7–8)	9 (8–9)	9 (8–10)
Delayed cord clamping*		56 (60%)	15 (57%)	18 (45%)	23 (34%)
Continuous Positive Airway Pressure*		40 (42%)	2 (8%)	16 (48%)	22 (63%)
Positive Pressure Ventilation*		54 (58%)	24 (92%)	17 (52%)	13 (37%)

Data are presented as mean±SD, unless indicated *n (%) or ^#^median (IQR).

**Table 2:** Systolic blood pressure measurements of term and preterm infants.

Time after birth	Term Infants (n = 54)	Preterm Infants (n = 94)
1 min		59 (53–66)
2 min		53 (47–64)
3 min	62 (60–66)	54 (47–59)
4 min	54 (37–64)	53 (47–62)
5 min	63 (55–73)	52 (47–59)
6 min	74 (61–80)	56 (50–63)
7 min	65 (59–71)	54 (48–58)
8 min	74 (66–78)	52 (48–63)
9 min	72 (63–73)	54 (49–63)
10 min	62 (58–68)	54 (49–62)
11 min	65 (55–75)	54 (47–59)
12 min	67 (56–72)	51 (48–60)
13 min	61 (60–72)	53 (46–61)
14 min	66 (62–72)	52 (48–60)
15 min	63 (59–69)	51 (47–57)
20 min		49 (43–54)
25 min		50 (42–54)
30 min		47 (42–53)
45 min		47 (42–53)
60 min		46 (40–48)

Data are presented as median (IQR).

**Table 3:** Diastolic blood pressure measurements of term and preterm infants.

Time after birth	Term Infants (n-54)	Preterm Infants (n = 94)
1 min		37 (25–40)
2 min		35 (25–40)
3 min	42 (35–45)	30 (27–36)
4 min	29 (24–35)	32 (28–37)
5 min	38 (33–46)	32 (27–35)
6 min	38 (24–58)	33 (27–34)
7 min	37 (26–42)	32 (27–35)
8 min	36 (32–42)	31 (26–36)
9 min	34 (28–37)	33 (29–36)
10 min	35 (28–38)	29 (26–36)
11 min	35 (24–36)	30 (26–36)
12 min	35 (30–45)	28 (25–39)
13 min	31 (24–40)	30 (23–35)
14 min	39 (28–45)	28 (24–33)
15 min	34 (29–40)	29 (24–35)
20 min		28 (24–34)
25 min		28 (22–33)
30 min		29 (23–33)
45 min		27 (21–32)
60 min		24 (21–27)

Data are presented as median (IQR).

**Table 4:** Mean blood pressure measurements of term and preterm infants.

Time after birth	Term Infants (n = 54)	Preterm Infants (n = 94)
1 min		47 (35–49)
2 min		41 (33–49)
3 min	49 (42–52)	38 (35–46)
4 min	39 (27–41)	40 (36–44)
5 min	46 (37–54)	39 (35–45)
6 min	43 (41–65)	39 (35–44)
7 min	49 (38–51)	39 (34–45)
8 min	49 (45–52)	38 (35–45)
9 min	46 (40–52)	40 (36–45)
10 min	44 (39–48)	38 (33–49)
11 min	40 (36–44)	38 (34–44)
12 min	46 (41–49)	38 (34–46)
13 min	45 (36–49)	37 (34–43)
14 min	52 (40–54)	36 (31–41)
15 min	44 (41–49)	36 (32–41)
20 min		36 (31–40)
25 min		36 (30–40)
30 min		35 (30–40)
45 min		33 (29–39)
60 min		30 (28–33)

Data are presented as median (IQR).

### Term infants

**Figure 2:**
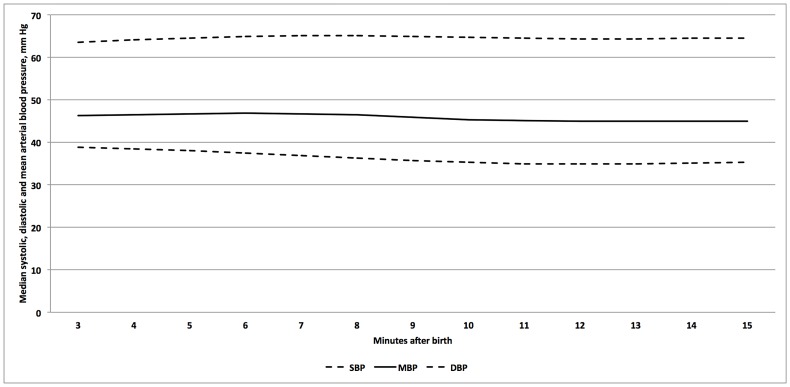
Median SBP, DBP, and MBP in mm Hg over the first 15 minutes after birth in term infants.

All term infants were delivered by cesarean section, none had DCC of 60 sec, and 10 (19%) of 54 required respiratory support (8/10 with CPAP, 2/10 with PPV; 1/10 required intubation and mechanical ventilation within the first 15 minutes and was admitted to the Neonatal Intensive Care Unit). The remaining term infants were given to the mothers for kangaroo care. The first BP measurements were obtained in the third minute after birth, after the baby was brought to the resuscitation table and the BP cuff was applied. There was no significant difference in SBP, DBP and MBP between infants who required respiratory support and those who did not. In addition, no gender differences were observed. Data from all term infants were therefore analyzed together. Median values of SBP, DBP, and MBP for all term infants are presented in Fig. 2.

### Preterm infants

**Figure 3:**
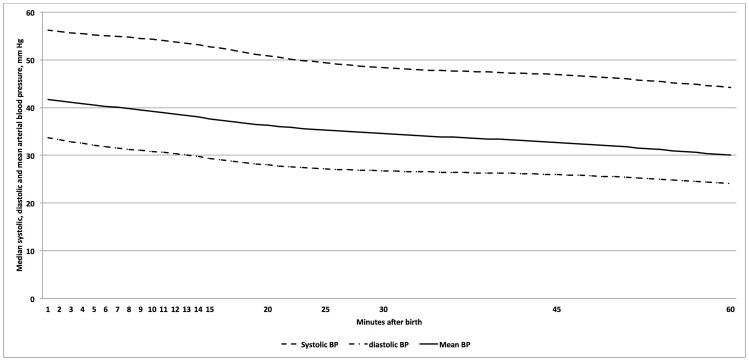
Median SBP, DBP, and MBP in mm Hg over the first 60 minutes after birth in preterm infants.

Of 94 preterm infants, 65 (74%) were born via cesarean section, 56 (60%) received DCC for 60 seconds, and all received respiratory support in the delivery room (54 (57%) PPV and 40 (43%) CPAP). Of the 94 preterm infants 26 infants were 23^+0^–27^+6^ weeks, 33 infants were 28^+0^–31^+6^ and 35 infants were 32^+0^–36^+6^. Overall values of BP in preterm infants are presented in Tables 2–4. Fig. 3 shows the median values of SBP, DBP, and MBP for all preterm infants. None of the preterm infants required inotropes support in the delivery room, however, 18/94 preterm infants (15<28 weeks, two infants 28–32, and one infant >32 weeks) received cardiovascular support after admission to the neonatal intensive care unit.

### Gender differences

There were no significant differences in gestational age, birth weight, mode of delivery, DCC or antenatal steroids between male and female infants. We observed no significant difference in SBP, DBP or MBP between male and females except for a significantly higher SBP, DBP, and MBP in female preterm infants at 25 minutes after birth. In female and male infants, SBP at 25 minutes was 51±10 vs. 46±9 mmHg (p = 0.03), DBP was 29±9 vs. 25±7 mmHg (p = 0.05), and MBP was 37±9 vs. 33±8 mmHg (p = 0.04), respectively.

### Gestational age

**Figure 4:**
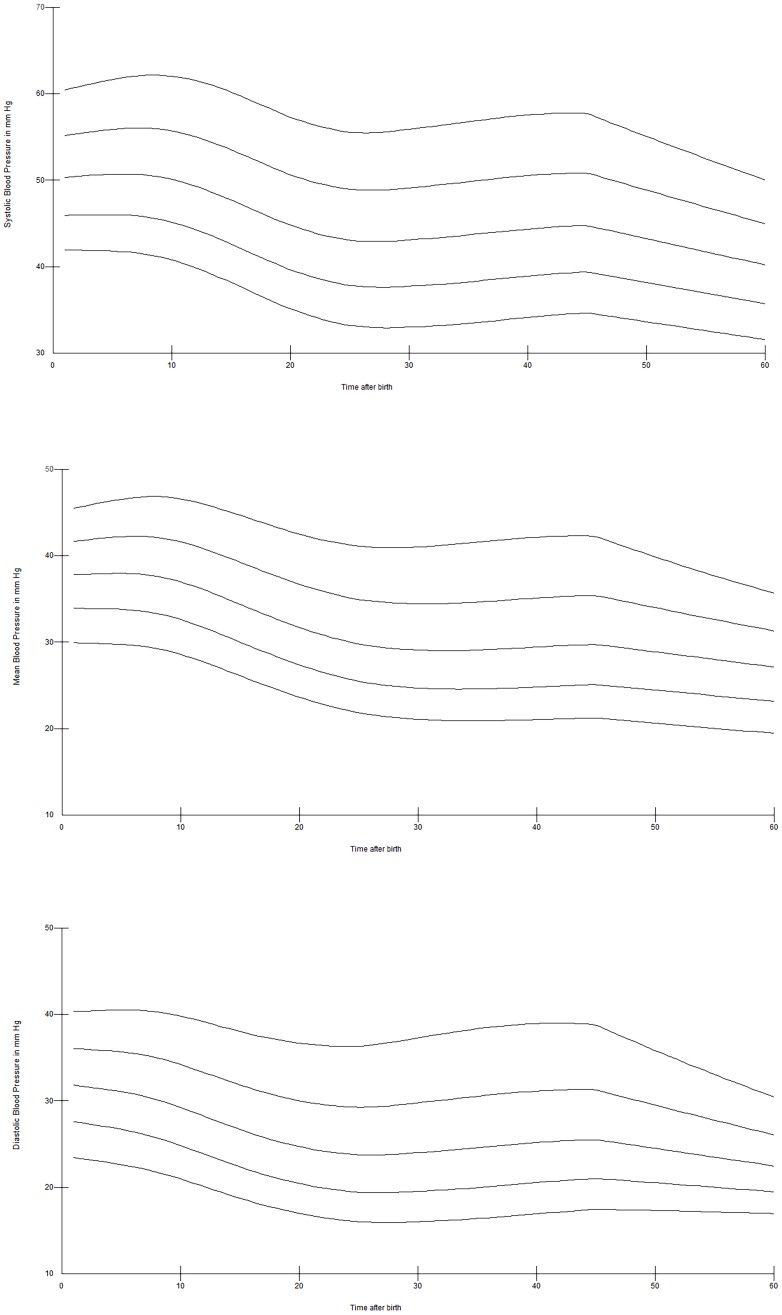
SBP (a), DBP (b), and MBP (c) centile over the first 60 minutes after birth in preterm infants 23^+0^–27^+6^ weeks gestation.

**Figure 5:**
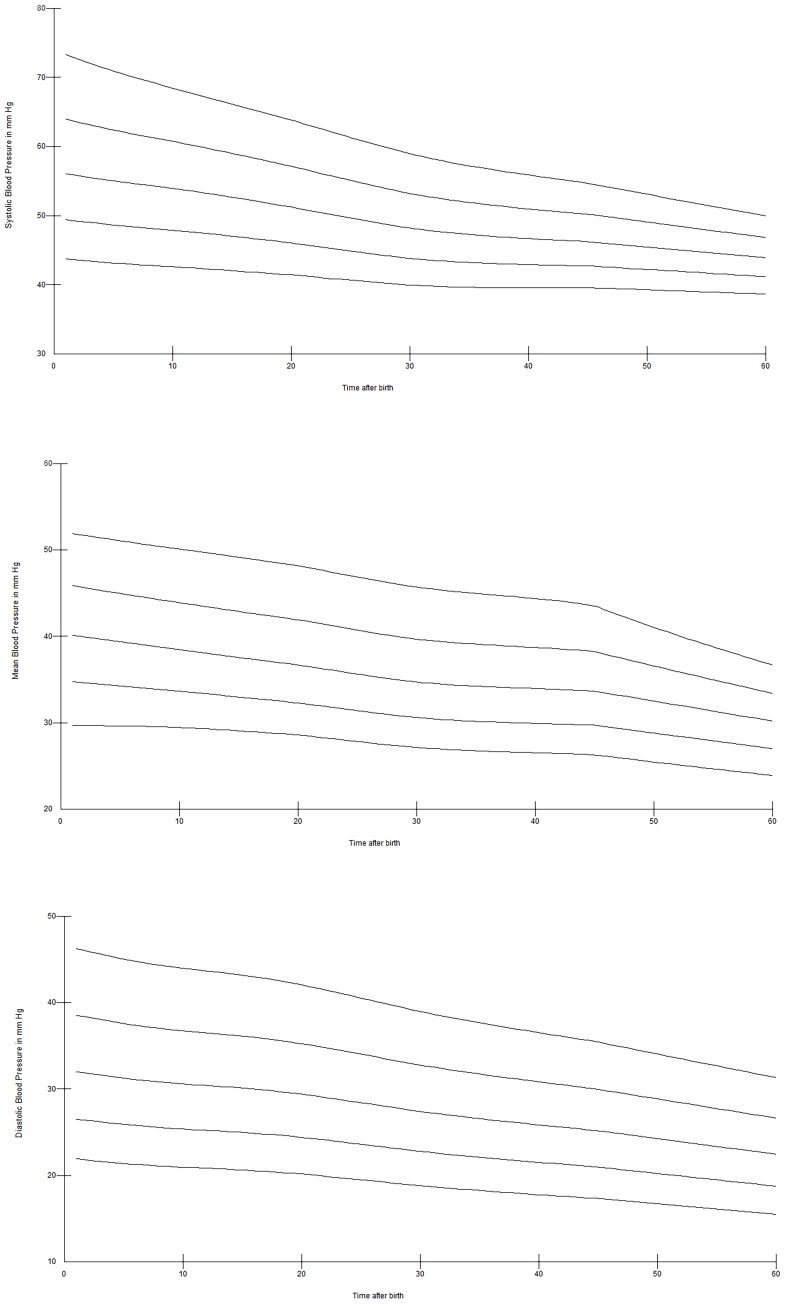
SBP (a), DBP (b), and MBP (c) centile over the first 60 minutes after birth in preterm infants 28^+0^–31^+6^ weeks gestation.

**Figure 6:**
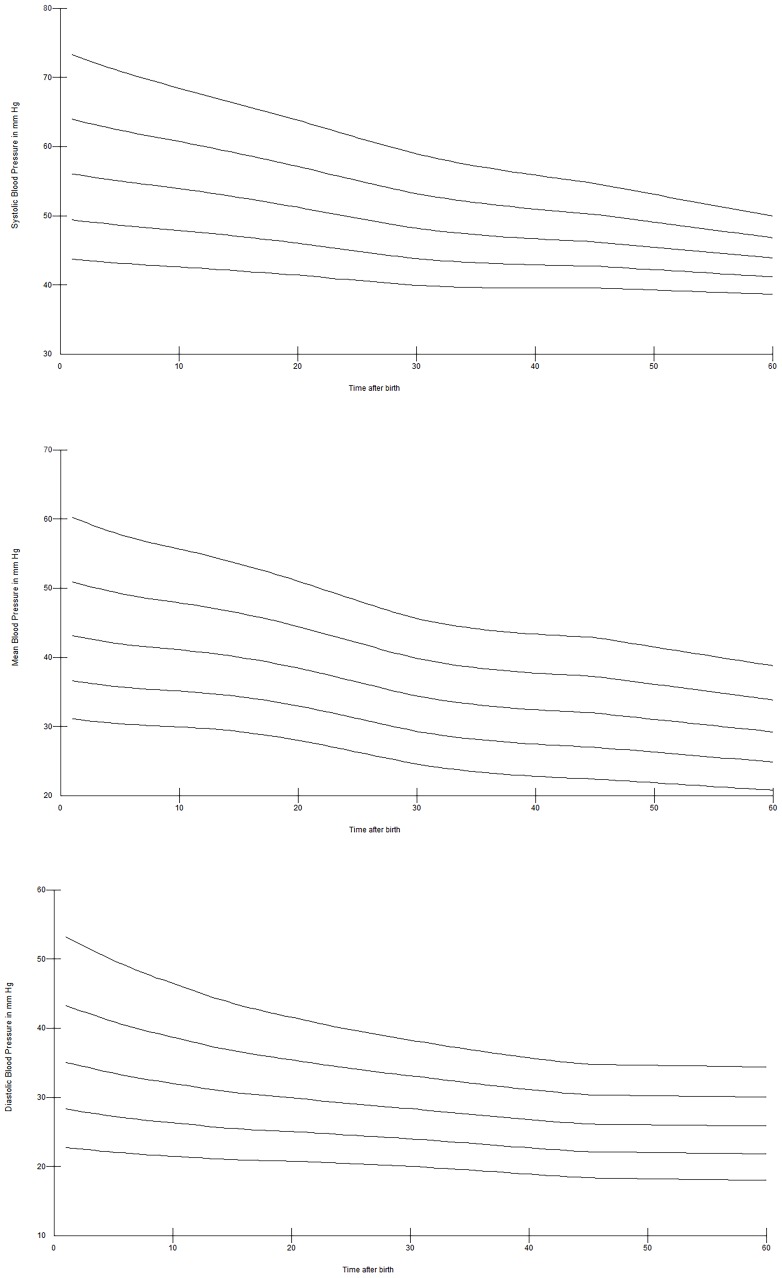
SBP (a), DBP (b), and MBP (c) centile over the first 60 minutes after birth in preterm infants 32^+0^–36^+6^ weeks gestation.

Twenty-six of 94 (28%) infants were born <28 weeks, 33 (35%) infants were born between 28^+0^–31^+6^ weeks, and 35 (37%)≥32 weeks gestation. Significantly more infants born <32 weeks received antenatal steroids 51/59 vs. 18/35 (p = 0000.1), respectively. There were no significant differences in mode of delivery and DCC between infants born <28, between 28^+0^–31^+6^ weeks and ≥32 weeks gestation. Centiles of SBP, DBP, and MBP for preterm infants born <28 weeks are presented in Fig. 4, for infants born 28^+0^–31^+6^ weeks in Fig. 5 and, for infants born ≥32 weeks gestation in Fig. 6. Analysis showed significantly higher SBP (at 1, 2, 3, 4, 10, 11, 12, 14, 20, 25, 30, and 60 minutes), MBP (at 1, 2, 3, 11, 12, 14, 20, 25, 30, 60 minutes) and DBP (at 1, 20, 30, 60 minutes) in preterm infants ≥32 weeks gestation compared to <28 weeks infants, all p<0.05. Infants 28^+0^–31^+6^ weeks had significant higher SBP at 1, 2, 5, 14, 20, and 25 minutes, MBP at 1 and 20 minutes, and DBP at 1 minute compared to <28 weeks infants (p<0.05). Comparison of infants 28^+0^–31^+6^ weeks vs.>32 weeks showed significant higher SBP at 1, 30 and 60 minutes, MBP at 1, 30 and 60 minutes, and DBP at 30 and 60 minutes in infants with >32 weeks (p<0.005).

### Mode of delivery

There were no significant differences in gestational age, birth weight, DCC or antenatal steroids between vaginal and cesarean delivery. Regression model showed BP was significantly higher at 7 (SBP), 8 (SBP and MBP), 9 minutes (MBP), and 45 minutes (SBP, DBP and MBP) after vaginal birth compared to caesarean section. SBP of vaginally born infants was 52±11 mmHg at 45 minutes, compared to 46±6 mmHg of those born by cesarean section, p = 0.009. DBP was also significantly higher at 45 min in vaginally born infants than that of infants born by caesarian section (32±13 vs. 25±7 mmHg, p = 0.01, respectively). MBP was also significantly higher at 45 minutes in vaginally delivered infants with 39±13 mmHg compared to 32±6 mmHg of infants born by caesarean section (p = 0.008). However, even though there is a statistically significant difference between the premature infants receiving DCC versus ECC, it is important to note that they were assisted at two different centers, with an unknown-and possibly differential-selection criteria and with different standards of care, which is a limitation of the study.

### Delayed cord clamping

DCC for 60 seconds was only performed in one center (Edmonton) with a total of 56 (60%) preterm infants (14 infants were <28 weeks, 21 infants were 28–32 weeks, and 20 infants were >32 weeks). There were no significant differences in gestational age, birth weight, mode of delivery, gender or antenatal steroids between infants receiving DCC and ECC. At 60 minutes after birth MBP and DBP was significantly higher in infants receiving DCC for 60 seconds with 33±6 mmHg and 26±6 mmHg vs. 28±4 mmHg and 22±5 mmHg, p = 0.01, and p = 0.04, respectively. No significant differences in SBP were observed within the first hour.

### Respiratory support

There were no significant differences in mode of delivery, DCC, gender, or antenatal steroids between groups who required different levels of respiratory support. However, infants receiving PPV had significantly lower gestational age and birth weight compared to infants receiving CPAP 28.5±3.4 weeks vs. 31.3±2.5 weeks (p<0.001) and 1169±667 grams vs. 1567±625 grams (p = 0.041), respectively. Although, regression analysis showed significant higher DBP and MBP (p = 0.0001 and p = 0.001) in preterm infants receiving CPAP at 60 minutes after birth compared to PPV. T-test comparison had similar SBP, DBP and MBP values throughout the first 60 minutes after birth.

### Ultrasound-detected brain injury

All preterm infants had cranial ultrasound prior to discharge and 13/94 (14%) had intraventricular hemorrhage grade 3 or 4 or a periventricular ischemic lesion (n = 4). Multiple regression analysis did not show any difference, however t-test comparison showed a significant lower SBP, and MBP at 25, 30 and 60 minutes of age and DBP 25 and 60 minutes in infants who subsequently developed intraventricular hemorrhage during their hospital admission. SBP at 25, 30 and 60 minutes in the infants developing intraventricular hemorrhage was 40±11 mmHg, 41±3 mmHg, and 38±6 mmHg compared to 50±9 mmHg, 49±7 mmHg, and 46±6 mmHg, p = 0.0005, p = 0.0008, and p = 0.001 respectively. MBP was 28±11 mmHg, 30±4 mmHg, and 26±3 mmHg compared to 36±7 mmHg, 36±6 mmHg, and 32±5 mmHg, p = 0.003, p = 0.01, and p = 0.002, respectively. DBP was 22±11 mmHg and 21±2 mmHg compared to 25±6 mmHg and 29±5 mmHg, p = 0.02 and p = 0.02, respectively.

## Discussion

To our knowledge this is the first study describing SBP, DBP, and MBP reference values in term and preterm infants immediately after birth. Overall, SBP, DBP, and MBP remained similar within the first 15 minutes after birth in term infants and decreased throughout the first 60 minutes after birth in preterm infants. Preterm infants, who were born vaginally, were female, had DCC for 60 seconds, and required CPAP had significantly improved SBP, DBP, and MBP values at some measurement points. Furthermore, preterm infants developing severe brain injury had significantly lower SBP, DBP, and MBP at 25, 30, and 60 minutes after birth.

Although, our results demonstrate that a low BP immediately after birth might requires cardiovascular support, however, the clinical condition of the infant has to be taken into account and this should be investigated in future studies. In addition, we report on physiological changes at birth and their clinical impact has to be defined in randomized studies.

### Term infants

Only term infants after elective caesarian section were included and therefore mode of delivery comparison was not possible, which is a limitation of the study. Infants, were not excluded from analyses, who needed cardio circulatory support after the first hour. Exclusion of neonates, in whom we don’t know yet if the blood pressure in the first hour after birth is normal or not, would have caused a bias in overall analyses of data. In addition, ethic approval was only granted for elective caesarian section and therefore we were unable to recruit infants born after vaginal delivery. Furthermore, we only measured for the first 15 minutes after birth as term infants were returned to their parents for kangaroo-care at that time point. Therefore we don’t know if BP changes afterwards.

### Preterm infants

We observed a steady decrease in SBP, DBP, and MBP throughout the first hour after birth. At first glimpse, this contradicts several echocardiography studies reporting a steady increase in stroke volume and CO in term infants within 1 hour after birth [4], [6]–[8]. However observations of BP, being a composite of both CO and SVR, may be explained by ductal flow and/or blood vessel relaxation and therefore blood redistribution.

### Mode of delivery

We observed a significant higher SBP, DBP, and MBP at 45 minutes after vaginal birth compared to caesarian section delivery. Our observations are similar to published data on peripheral oxygen saturation (from 1 until 5 minutes) and heart rate (from 2 to 10 minutes), which reported higher values after vaginal delivery; in comparison published data on cerebral oxygenation was similar regardless of mode of delivery during neonatal transition[9]–[13]. Observational studies in vaginally born term infants reported an increased left stroke volume over the first 15 minutes, whereas right stroke volume was unchanged [2]. In addition, *Walther et al* and *Agata et al* reported that CO peaked between 30 minutes to 2 hours after birth with a steady decreased to reach a steady state thereafter [4], [7].

### Delayed Cord Clamping

DCC is believed to improve cardiovascular function and stability during the immediate transition to neonatal life after birth. DCC for 60 seconds can increase an infant’s blood volume by approximately 16 mL/kg and reduce rates of intraventricular hemorrhage in premature infants [14]–[16]. Some of the benefits of DCC are due to the circulatory transition initiated by air breathing [17], as well as due to improved circulating blood volume, including brain blood flow resulting in increased blood flow in the superior vena cava. In the current study MBP and DBP, but not SBP, was significantly increased 60 minutes after birth in the DCC group compared to the ECC group. Nevertheless the present comparison of DCC and ECC must be interpreted with caution, although DCC was routinely performed for 60 seconds after delivery, it was not randomized or not performed if the obstetric team noted contraindications (e.g. antepartum hemorrhage, fetal/neonatal bradycardia or apnea). In 51 preterm infants, *Sommers et al* [19] evaluated hemodynamic effects of DCC vs. ECC at 48 hours of life. Infants exposed to DCC had higher blood flow through the superior vena cava, increased right ventricular output, and right ventricular stroke volumes. In addition, infants exposed to DCC trended to have a higher right ventricular stroke volume, however no differences in shortening fraction compared to ECC was identified [18]. In a small group of healthy term infants DCC of 4 minutes resulted in improved venous return and left ventricular function on day 3 of age compared to immediately clamped infants [19]. These improvements are most likely redistribution from the capillary blood toward alternative vascular beds, preventing a relative ischemia and hypovolemia and have the potential to improve neonatal adaptation. However, *Bhatt et al* recently described that ventilation prior to umbilical cord occlusion improved cardiovascular function and stability during the immediate transition to neonatal life after birth in preterm lambs [17]. This indicates that lung aeration mitigated most of the adverse cardiovascular responses to cord clamping [17]. In comparison, early clamping causes a significant decrease in pulmonary vascular resistance, reduced blood volume and has a profound influence on cardiovascular function after birth [17], [20].

### Respiratory Support

Lung aeration at birth results in an 8–10 times increase in pulmonary blood flow, which is essential for both pulmonary gas exchange and maintenance of left ventricular output [3], [21], [22]. A recent study by *Bhatt et al* demonstrated that infants receiving ventilation before DCC had significantly improved hemodynamics [17]. In addition, a study by *Binder et al* reported no differences in BP-values within the first 10 minutes after birth [22], which is similar to our findings. One study that examined hemodynamic changes after surfactant administration in the delivery room reported an increase in right ventricular output but decrease in left ventricular output after surfactant administration [23].

In the current study we had similar BP values for infants requiring CPAP or PPV. Complicated effects of intrathoracic pressure and cardiopulmonary interaction in the transitional circulation may explain the lack of significant difference between groups who required different levels of respiratory support.

### Preterm brain injury

Our data show that preterm babies who have severe brain injury on cerebral ultrasound have significantly different cardiovascular adaptation at birth, with lower BP readings at 25 to 30 minutes and at 60 minutes of age. It is difficult to know whether these findings reflect the possibility that injury starts prior to delivery, or that injury may be modified by interventions that occur during early transition, such as DCC or ventilatory support. Since the present study is not designed and powered to answer any causal relationships of blood pressure and brain injury, it would be prudent to choose interventions that improve BP stability.

### Limitations

Observational studies such as this are limited by patient selection, however, the bias was mitigated to some extent by our larger patient numbers, more than one delivering institution, and prospective inclusion of potential confounders, such as gender and mode of delivery. Measurement of BP may vary considerably with the underlying methodology and the BP cuff size, which potentially are confounder in measurement of peripheral BP. However, these methodologies for ascertaining BP are in common use in intensive care units around the world, giving our reference ranges clinical generalizability. In addition, the non-invasive BP recording has its limitations and correlates poorly with invasive BP [24]. A more recent study comparing invasive and non-invasive found a good agreement between oscillometric and invasive reading with the exception blood pressure at the lower limit [25]. These findings emphasize the necessity of careful evaluation of low blood pressure in preterm neonates. We did not perform echocardiography or peripheral blood flow measurements, which would have added substantial information to the cardiovascular changes during neonatal transition after birth and is a limitation to the current study. Echocardiography might have identified changes in CO, whereas measurements of peripheral blood flow using near-infrared spectroscopy or laser doppler flow study [26] might have revealed changes in systemic vascular resistance, regional blood flow, or oxygen extraction. Newer point-of-care technologies will hopefully address these gaps in future. It will be interesting to determine why BP continuously decreases during the first minutes after birth.

## Conclusions

Non-invasive measurements of blood pressure are feasible during immediate neonatal transition and provide important information on the cardiovascular status of the newborn. These are the first reference ranges of BP in a cohort of term and preterm neonates. We have formulated centile charts that may be used to optimization cardiovascular support during immediate transition in both term and preterm infants.
